# Apolipoprotein E genotypes among diverse middle-aged and older Latinos: Study of Latinos-Investigation of Neurocognitive Aging results (HCHS/SOL)

**DOI:** 10.1038/s41598-018-35573-3

**Published:** 2018-12-13

**Authors:** Hector M. González, Wassim Tarraf, Xueqiu Jian, Priscilla M. Vásquez, Robert Kaplan, Bharat Thyagarajan, Martha Daviglus, Melissa Lamar, Linda C. Gallo, Donglin Zeng, Myriam Fornage

**Affiliations:** 1 Department of Neurosciences and Shiley-Marcos Alzheimer’s Disease Research Center, University of California, San Diego, La Jolla, CA USA; 2 Institute of Gerontology & Department of Healthcare Sciences, Wayne State University, Detroit, Michigan USA; 3 Institute of Molecular Medicine, McGovern Medical School University of Texas Health Science Center, Houston, TX USA; 4 Albert Einstein, College of Medicine, New York, USA; 5 Department of Laboratory Medicine and Pathology, University of Minnesota Medical Center Fairview, Minneapolis, Minnesota USA; 6 Institute for Minority Health Research, University of Illinois at Chicago, College of Medicine, Chicago, Illinois USA; 7 Rush University, Chicago, IL USA; 8 Institute for Behavioral and Community Health, Graduate School of Public Health, San Diego State University, San Diego, California USA; 9 Department of Biostatistics, Collaborative Studies Coordinating Center, University of North Carolina, Chapel Hill, North Carolina USA; 10 Human Genetics Center, School of Public Health, University of Texas Health Science Center, Houston, TX USA

## Abstract

The apoE4 isoform is associated with increased cholesterol, cardiovascular risk, and Alzheimer’s Disease risk, however, its distribution is not well-understood among US Latinos. Latinos living in the US are highly Amerindian, European and African admixed, which varies by region and country of origin. However, Latino genetic diversity is understudied and consequently poorly understood, which has significant implications for understanding disease risk in nearly one-fifth of the US population. In this report we describe apoE distributions in a large and representative sample of diverse, genetically determined US Latinos.

## Introduction

The apolipoprotein E (apoE) gene encodes three common isoforms known as ε2, ε3, and ε4. These are determined by two single nucleotide polymorphisms (SNPs) that result in amino acid substitutions and associated functional changes in the protein. The apoE4 isoform is associated with increased circulating levels of total cholesterol and low-density lipoprotein (LDL), cardiovascular risk, Alzheimer’s Disease (AD), and related dementias (ADRD)^1–5^; whereas apoE2 is associated with cognitive resilience and extended longevity^6^. Given its broad impact on health and disease, it is important to characterize the distribution of this significant genetic risk and possibly protective factor among diverse and understudied populations, including Hispanics/Latinos (henceforth Latino). Much of the genetic epidemiologic literature on Latino apoE distributions comes from small anthropologic studies in Latin America^7–9^. More recent studies of apoE and ADRD are limited to samples of older Latino adults of Mexican, Puerto Rican, or Dominican-origin. These are generally small and relatively homogenous samples that may be affected by selection and survival biases^1,10–12^. As such, it is difficult if not impossible to cogently describe the distribution of apoE alleles among Latinos without examining the diversity of Latinos. In this brief report, we examine new apoE genotype information on 10,887 Latino adults of diverse and well-defined ancestry background.

## Results

The characteristics of the sample are shown in Table 1. A total of 10,887 Hispanic Community Health Study/Study of Latinos (HCHS/SOL) participants of diverse genetically-determined Latino backgrounds were genotyped for apoE. Slightly more than half of the target population (50.4%) was female, and the average age was 41.5 years. The distribution of the self-reported background groups was as follow: Mexicans were the largest group (33.9%), followed by Cubans (22.8%), Puerto Ricans (16.3%), Dominicans (10.5%), Central Americans (7.2%), and South Americans (5%). Compared to participants without an apoE genotype, the genotyped sample was slightly older and more educated; had a lower proportion of women and Mainland Latinos (Mexicans, Central Americans and South Americans); and a higher proportion of Caribbean Latinos (Cubans, Dominicans, and Puerto Ricans). Among unrelated participants with apoE genotype, there were statistically significant Latino background differences in age and sex. Cubans (47.1 years) were the oldest group, followed by Puerto Ricans (43.0-years), South Americans (42.2 years), Central Americans (40.2 years), Dominicans (39.9 years) and Mexicans (39.1 years). Secondly, Dominicans had the highest proportion of females (61.1%), followed by South Americans (52.2%), Mexicans (48.3%), Puerto Ricans (47.0%), Central Americans (46.9%) and Cubans (46.4%). The group differences were generally small and do not suggest sampling bias.

**Table 1:** Characteristics of the SOL-INCA sample genotyped for apoE and comparisons with those without apoE genotype.

	With ApoE genotype	No ApoE genotype	P value
N	10,887	5,472	
Age (95% CI), years	41.4 (41.0–41.8)	40.2 (39.6–40.8)	0.014
Women, %	50.4	55.9	<0.0001
Genetic Ancestry Group, %			
Central American	7.7	9.4	<0.0001
Cuban	24.8	19.8	
Dominican	10.5	6.7	
Mexican	34.1	43.6	
Puerto Rican	17	14.1	
South American	5.9	6.3	
Education, %			
Less than high school	31	35.3	0.0004
High school or equivalent	28.3	27.8	
Greater than high school	40.6	36.8	

CI: confidence interval.

Within genetic ancestry group, allele frequencies of rs429358 and rs7412, which determine the apoE genotype, did not deviate from Hardy-Weinberg expectations (Supplemental data). Table 2 presents apoE genotype distributions and frequency proportions of the apoE2, E3 and E4 alleles in each of the 6 genetic ancestry groups. Frequency of the apoE3 allele ranged from 73.9% in Dominicans to 86.2% in Mexicans. Frequency of the apoE4 allele was highest in Dominicans (17.5%) and lowest in Central Americans, South Americans and Mexicans (~11%), while Cubans and Puerto Ricans were slightly higher (12.6% and 13.3%, respectively). Mexicans had the lowest ApoE2 allele frequency (2.9%). South and Central Americans were slightly higher (3.6% and 3.9%, respectively) and these estimates were twice as high in Cubans (6.5%) and Dominicans (8.6%). The apoE22 genotype was not observed in our sample of South Americans while the apoE44 genotype was more than twice as frequent in Dominicans (3.6%) as in Mexicans and Central Americans (1.2%).

**Table 2:** ApoE genotype and allele frequency proportions in percent (95% confidence interval) among unrelated HCHS/SOL participants by genetic ancestry group.

	Central American	Cuban	Dominican	Mexican	Puerto Rican	South American	P-value (χ^2^)
	N = 931	N = 1679	N = 725	N = 2900	N = 1416	N = 612	
**Genotype**							
E22	0.32(0.01; 0.69)	0.48(0.15; 0.81)	0.69(0.09; 1.29)	0.1(0.01; 0.22)	0.35(0.04; 0.66)	0	
E23	6.02(4.49; 7.54)	10.9(9.41; 12.39)	12.83(10.39; 15.26)	5.07(4.27; 5.87)	8.04(6.62; 9.45)	6.37(4.44; 8.31)	
E24	1.07(0.41; 1.74)	1.13(0.63; 1.64)	3.03(1.79; 4.28)	0.45(0.21; 0.69)	1.69(1.02; 2.36)	0.82(0.01; 1.53)	
E33	72.93(70.08; 75.76)	65.52(63.24; 67.79)	55.17(51.55; 58.79)	74.1(72.51; 75.70)	66.57(64.12; 69.03)	73.37(69.86; 76.87)	
E34	18.47(15.98; 20.87)	19.95(18.04; 21.86)	24.69(21.55; 27.83)	19.03(17.61; 20.46)	21.72(19.57; 23.87)	17.32(14.32; 20.32)	
E44	1.18(0.49; 1.88)	2.03(1.35; 2.70)	3.59(2.23; 4.94)	1.24(0.84; 1.64)	1.62(0.96; 2.28)	2.12(0.98; 3.27)	<0.0001
**Allele**							
E2	3.87(2.99, 4.74)	6.49(5.66, 7.33)	8.62(7.18, 10.07)	2.86(2.43, 3.29)	5.22(4.40, 6.04)	3.59(2.55, 4.64)	
E3	85.18(83.56, 86.79)	80.94(79.61, 82.27)	73.93(71.67, 76.19)	86.16(85.27, 87.03)	81.45(80.02, 82.88)	85.21(83.22, 87.20)	
E4	10.96(9.57, 12.46)	12.57(11.45, 13.69)	17.45(15.49, 19.40)	10.98(10.18, 11.79)	13.33(12.08, 14.58)	11.19(9.43, 12.96)	<0.0001

Estimates of pairwise genetic distance among the 6 groups based on the two ApoE SNPs were close to 0 indicating no evidence of divergence between the groups, but significant permutation P-values likely reflect differences in ApoE allele distributions between the genetic ancestry groups (Table 3). Using self-reported ancestry information did not meaningfully affect these results (Supplemental data). Similarly, apoE allele frequency distributions were not significantly different between participants younger than age 50 years and those aged 50 years and older in any of the genetic ancestry groups (Supplemental Table 1) or in the overall sample (P = 0.34). The same conclusions were reached when using age groups younger than 60 years and 60 years and older (P = 0.87; Supplemental Table 2).

**Table 3:** Pairwise genetic distances (Slatkin linearized F_ST_ values) based on the two ApoE single nucleotide polymorphisms among SOL genetically-determined ancestry groups.

	Central American	Cuban	Dominican	Mexican	Puerto-Rican	South American
Central American	0					
Cuban	0.00259*	0				
Dominican	0.01806*	0.00711*	0			
Mexican	0.00004	0.00528*	0.02541*	0		
Puerto-Rican	0.00199*	0.00033	0.00721*	0.00373*	0	
South American	0	0.00388*	0.01989*	0	0.00287*	0

*Indicates a significant P value (P < 0.05) for the permutation test of the null hypothesis of no difference between the populations.

## Discussion

In this large sample of diverse Latinos living in four targeted US metropolitan areas, we found heterogeneous apoE allele frequency distributions by ancestry background. Frequency of the apoE4 allele, a risk factor for cognitive decline and ADRD, was highest among Caribbean Latinos, namely Dominican, Puerto Ricans and Cubans. The apoE4 allele frequency was lowest among Mainland Latinos - Central Americans, Mexicans and South Americans. Frequency of the apoE2 allele, a putative resilience factor for cognitive decline and ADRD, was highest among Caribbean Latinos and lower among Central Americans, with South Americans and Mexicans having the lowest frequencies. We did not find supportive evidence for differential apoE allele frequencies by age group either within ancestry groups or in the overall sample.

American human population origins arc over 15,000 years CE with repeated waves across the millennia of migrants to the North and South American continents, including more recent (~500 CE) but impactful European colonization and African forced labor that drastically altered the genetic make-up of populations in different regions in the Americas. Conomos and colleagues used genome-wide SNP data to estimate continental-ancestry proportions in HCHS/SOL participants based on assumptions of three ancestral populations, Amerindian, African and European. They described distinguishable patterns of continental-ancestry proportions among HCHS/SOL participants of Mainland and Caribbean backgrounds. Specifically, participants of self-identified Mexican, Central, and South American background (Mainlanders) have more Amerindian and less African ancestry than those of self-identified Caribbean background, with those of Dominican background having more African ancestry and those of Cuban background having more European ancestry than the other groups^13^. The distributions of apoE genotype and allele frequencies observed in this study were consistent with these reported continental-ancestry patterns and with previously reported anthropologic and genetic studies of populations from these three continents^7,14,15^: Mainland Latinos had lower apoE4 and apoE2 allele frequencies compared to Caribbean Latinos. ApoE2 and apoE4 allele frequencies were highest among Dominicans consistent with the known higher frequencies of these alleles reported from African populations^16^.

Studies in non-human primates and more recent examination of Neanderthal and Denisovan nuclear DNA suggest that apoE4 is the ancestral allele^16^. ApoE3 is the most frequent allele in all human populations ranging from 69–85% and its frequency correlates negatively with that of apoE4, indicating a progressive substitution of the ancestral allele with the new allele^7^. Various hypotheses have been proposed to explain the predominance of apoE3 over apoE4, including energy conservation age-related disease risk in post-reproductive humans, notably cognitive decline and ADRD^17^.

Latinos are more likely than Whites to develop AD and ADRD^18^. However, studies investigating the association of apoE4 with risk and onset of AD and AD-related traits in Latinos have not always produced consistent findings, likely reflecting the heterogeneity of Latino groups in which they were conducted. Several studies reported a low or absent apoE4-associated risk for AD^10,12^. However, these conclusions are not generalizable to all Latino groups. Many studies have been performed in samples recruited in the northeastern US and comprised of participants mostly of Dominican and other Caribbean origin. For example, Tang and colleagues reported apoE4 allele frequencies of “Hispanics” in the New York area (primarily Dominicans) over age 65-years (N = 188) that were similar for both normal (14.1%) and AD (14.8%) groups but were lower than our estimates^10^. They did not identify a significant association between apoE4 and elevated incident AD risk, but were likely underpowered to detect differences^10,19^. A more recent study of 203 families with at least 2 living relatives with a history of dementia recruited from the same Caribbean Latino communities in the greater New York City area, the Dominican Republic, and Puerto Rico reported a higher apoE4 allele frequency (23.2% in control group and 32.4% in the AD group) and a significant association of that allele with AD risk^20^. Although these reported allele frequencies were more congruent with our estimates, the familial AD sampling likely resulted in enrichment for the apoE4 allele. In a study in Cuban Americans living in South Florida (N = 80 cases and 21 controls), the apoE4 allele frequency was higher for AD patients than for controls without dementia (25% vs. 7.1%)^21^. Similarly, in a small sample of age- and education-matched “Mexican Hispanic” AD cases (N = 28) and dementia-free controls (N = 28) from Southern California, the estimated apoE4 allele frequency in AD cases (21.4%) was higher than controls (12.5%)^22^. In both studies, apoE4 frequency differences between cases and controls were not statistically significant likely due to low power. In the Sacramento Area Latino Study on Aging (SALSA) study of Mexican Americans over age 60-years from the north central valley of California, apoE4 was associated with increased dementia risk. In SALSA, the apoE4 allele frequency was much lower (4%) than in the present study and all previous studies we reviewed. The authors attributed their very low reported apoE4 allele frequency to lower survival of E4 homozygotes^23^. In a meta-analysis study that included SALSA, the apoE4 allele frequency (7.5%), which was more in line with SOL-INCA^24^. No explanations were provided for the different estimates. A Texas-based study of Mild Cognitive Impairment (MCI) in Mexican Americans (N = 626) found the frequency of apoE4 carriers to be 18% and 22%, respectively, in dementia-free individuals, similar to our estimates. However, there was no association of apoE4 with MCI in either of these studies of Mexican Americans^12^. Taken together, these data underscore the need for large studies of diverse Latinos with well-characterized ethnic backgrounds to better understand the nature of the association between AD and the apoE genotype in the fastest-growing segment of the US population.

Similar to other populations worldwide, apoE2 in SOL-INCA Latinos was the least common of the 3 major apoE alleles, ranging in frequency from 2.9% in Mexicans to 8.6% in Dominicans. This is in line with the absent or low apoE2 allele frequency observed in many South American native societies and the relatively high allele frequency in Africa (9.9%)^7^. ApoE2 is reportedly associated with reduced ADRD risk in other ethnic/racial populations. Similarly, apoE2 has been associated with increased longevity in European-ancestry populations, but not among Latinos. Paradoxically, Latino life-expectancy at birth exceeds that of Whites by 3-years despite adversities (e.g., low socioeconomic status) thought to reduce longevity and low E2 allele frequencies among most Latino. Indeed, there is little information about how E2 is associated with health, disease and life-expectancy among Latinos as a whole and among diverse Latinos.

Several studies have reported a lower frequency of the apoE4 allele in older age groups compared to younger or middle-aged individuals. The age at which a decline in apoE4 frequency is observed has varied across studies but most reported decrease in apoE4 after age 60^25,26^. In our sample of diverse mostly middle-aged Latinos, we did not observe differences in apoE allele frequency or apoE4 genotype distributions among participants older than 60 compared to those younger than 60. However, HCHS/SOL did not enroll participants beyond the age of 75 at baseline and, thus, we had little power to investigate apoE allele frequency distributions in older age groups.

This report has several strengths. To our knowledge, this is the first study to use Latino genetic ancestry, characterized based on genome-wide SNP data, in relation to apoE. The data used for this study includes the largest and most diverse sample, to-date, of US Latinos. Targeted metropolitan area representativeness was achieved using a complex sampling approach, which is unique in genetics research. However, we also note that our apoE allele frequency estimates are based on a sample of US participants ascertained from four urban communities, which may not be representative of the entire US Latino population or of populations in Latin-America from which they originate.

In conclusion, we report apoE allele and genotype frequency distributions in a large and diverse sample of Latinos with well-characterized ancestry background. These data provide valuable information in this understudied ethnic group and provide the basis for future studies of the association of apoE with ADRD in this fast-growing segment of the US population.

## Methods

### Study population

The data used in this study are from the Hispanic Community Health Study/Study of Latinos (HCHS/SOL). Written informed consent was obtained from all included participants, and the study was reviewed and approved by the Institutional Review Boards of UC San Diego and all collaborating institutions. HCHS/SOL participants were sampled to ensure generalizability of inferences to Latinos, ages 18–74 years at recruitment, in four major population areas in New York (Bronx), Illinois (Chicago), Florida (Miami) and California (San Diego). Sampling procedures incorporated six major Hispanic/Latino groups including: Dominicans, Central Americans, Cubans, Mexicans, Puerto Ricans, South Americans and oversampled participants 45-years and older. The data includes a rich collection of biological specimens (e.g. blood) in addition to detailed demographic, sociocultural, and health histories. The study objectives, design, sampling procedures and implementation, and a detailed discussion of the data modules have been previously published^27,28^.

### ApoE genotyping

ApoE genotyping was performed as part of the Study of Latinos - Investigation of Neurocognitive Aging (SOL-INCA), a HCHS/SOL ancillary study of cognitive aging, Mild Cognitive Impairment (MCI) and, to a lesser extent, dementia. Genotyping of SNPs rs429358 and rs7412 was performed using pre-formulated TaqMan allele-discrimination assays (assay IDs: C___3084793_20 and C____904973_10) (commercialized by Thermo Fisher Scientific, Waltham, MA). For each polymorphism, a PCR product was amplified utilizing 0.9 μM each of the forward and reverse primer, 0.2 μM each of sequence-specific probes, 3 ng DNA and 1X TaqMan Universal PCR Master Mix in a 6 µl reaction volume. After an initial step of 2 min at 50 °C and 10 min at 95 °C, the products were amplified using 40 cycles of 15 s at 95 °C and 1 min at 62 °C. Allele detection and genotype calling were performed using the ABI 7900HT and the Sequence Detection System software (Applied Biosystems, Foster City, CA).

### Genetically-determined ancestry background

Genome-wide SNP genotyping was performed on 12,803 HCHS/SOL participants using a custom Illumina (San Diego, CA) array consisting of the HumanOmni2.5–8v1–1 array content along with a panel of ∼150 000 investigator-chosen SNPs. These data were used to assign each participant to one of 6 genetic analysis groups defined as having the same six values as the self-identified ancestry background groups (i.e., Cuban, Dominican, Puerto Rican, Mexican, Central American, and South American). Details of the methodology have been previously published^13^. Briefly, the method uses a multi-dimensional clustering algorithm to construct a categorical variable (“genetic-analysis group”), designed to be similar to self-identified background groups with regards to cultural and environmental characteristics but to be more genetically homogeneous, as determined by Principal Component analysis. There was a high concordance between self-identified ancestry and genetic ancestry (94–98% for each of the six ancestry groups). A group of 37 individuals mostly Central Americans with unusual ancestry were excluded from the definition of genetic-analysis groups and therefore from these analyses^13^.

### Statistical Analyses

Analyses were carried out using SAS version 9.4 (SAS Institute Inc, Cary, NC). Descriptive sample statistics were carried out in the total genotyped sample with available genetic ancestry group data (N = 10,887) and were weighted to adjust for sampling probability and nonresponse as previously described^27^. All other analyses were carried out excluding related individuals based on the calculated pairwise kinship coefficients and using a threshold value of 0.025 (N = 2,622 relatives excluded)^13^. Allelic and genotypic frequencies were estimated by gene counting within genetic ancestry groups. Differences in genotype or allele frequencies between genetic ancestry groups were evaluated using chi-square tests. Hardy-Weinberg equilibrium tests were computed for each SNP within genetic ancestry group and departure from expectations were evaluated using a chi-square test.

We also calculated the pairwise Slatkin linearized F_ST_ indices among genetically-determined ancestry groups using the Arlequin software (v.3.5.2.2)^29^. These were used as a measure of short-term genetic distances between the 6 Latino groups^30^. A permutation test assessing the hypothesis of no difference between groups was performed by permuting apoE haplotypes.

## Electronic supplementary material

Supplementary information
