# Supplementary material for: Apolipoprotein E genotypes among diverse middle-aged and older Latinos: Study of Latinos-Investigation of Neurocognitive Aging results (HCHS/SOL)
Source: Sci Rep. 2018 Dec 13;8:17578. doi: 10.1038/s41598-018-35573-3 (PMC6292877; doi:10.1038/s41598-018-35573-3)
Supplement: Supplementary file 1 — Supplementary information [file 41598_2018_35573_MOESM1_ESM.doc]

**Apolipoprotein E genotypes among diverse middle-aged and older Latinos: Study of Latinos-Investigation of Neurocognitive Aging results (HCHS/SOL)**

Hector M. González,^1^ Wassim Tarraf,^2^ Xueqiu Jian,^3^ Priscilla M. Vásquez,^1^ Robert Kaplan,^4^ Bharat Thyagarajan,^5^ Martha Daviglus,^6^ Melissa Lamar,^7^ Linda C. Gallo,^8^ Donglin Zeng,^9^ Myriam Fornage^3, 10^

^1^ Department of Neurosciences and Shiley-Marcos Alzheimer’s Disease Research Center, University of California, San Diego, La Jolla, CA

^2^ Institute of Gerontology & Department of Healthcare Sciences, Wayne State University, Detroit, Michigan

^3^ Institute of Molecular Medicine, McGovern Medical School University of Texas Health Science Center, Houston, TX

^4^ Albert Einstein, College of Medicine

^5^ Department of Laboratory Medicine and Pathology, University of Minnesota Medical Center Fairview, Minneapolis, Minnesota

^6^ Institute for Minority Health Research, University of Illinois at Chicago, College of Medicine, Chicago, Illinois

^7^ Rush University, Chicago, IL

^8^ Institute for Behavioral and Community Health, Graduate School of Public Health, San Diego State University, San Diego, California

^9^ Department of Biostatistics, Collaborative Studies Coordinating Center, University of North Carolina, Chapel Hill, North Carolina

^10^ Human Genetics Center, School of Public Health, University of Texas Health Science Center, Houston, TX

*Corresponding Authors:

**Hector M. Gonzalez, PhD, Department of Neurosciences**

University of California, San Diego, 9500 Gilman Dr. La Jolla, CA 92093-0948. Phone: (858) 534-5361. Fax: (858) 534-8242.

Email: [hectorgonzalez@ucsd.edu](mailto:hectorgonzalez@ucsd.edu)

**Myriam Fornage, PhD, Department of Epidemiology**

University of Texas Health Science Center, 1200 Pressler Street, Houston, TX 77030. Phone: (713) 500-9058.

Email: [myriam.fornage@uth.tmc.edu](mailto:myriam.fornage@uth.tmc.edu)

**Supplemental Table 1.** ApoE genotype and allele frequency distribution (in percent) by age category and genetic ancestry group among unrelated HCHS/SOL participants

|  | Central American | | Cuban | | Dominican | | Mexican | | Puerto-Rican | | South American | |
| --- | --- | --- | --- | --- | --- | --- | --- | --- | --- | --- | --- | --- |
|  | < 50  N=552 | 50 +  N=379 | < 50  N=807 | 50 +  N=872 | < 50  N=406 | 50 +  N=319 | < 50  N=1730 | 50 +  N=1170 | < 50  N=699 | 50 +  N=719 | < 50  N=324 | 50 +  N=288 |
| Genotype | | | | | | | | | | | | |
| apoE4- | 79.89 | 78.36 | 77.20 | 76.61 | 70.69 | 66.14 | 78.38 | 80.60 | 74.82 | 75.10 | 81.48 | 77.78 |
| apoE4+ | 20.11 | 21.64 | 22.80 | 23.39 | 29.31 | 33.86 | 21.62 | 19.40 | 25.18 | 24.90 | 18.52 | 22.22 |
| P-value (χ2) | 0.57 | | 0.77 | | 0.19 | | 0.14 | | 0.90 | | 0.26 | |
| Allele | | | | | | | | | | | | |
| E2 | 3.99 | 3.69 | 6.63 | 6.36 | 7.88 | 9.56 | 2.86 | 2.86 | 5.29 | 5.15 | 3.09 | 4.17 |
| E3 | 85.60 | 84.56 | 80.67 | 81.19 | 75.49 | 71.94 | 85.64 | 86.92 | 81.40 | 81.50 | 86.88 | 83.33 |
| E4 | 10.42 | 11.74 | 12.70 | 12.44 | 16.83 | 18.50 | 11.50 | 10.21 | 13.30 | 13.35 | 10.03 | 12.50 |
| P-value (χ2) | 0.64 | | 0.92 | | 0.29 | | 0.30 | | 0.98 | | 0.21 | |

**Supplemental Table 2.** ApoE genotype and allele frequency distribution (in percent) by age category and genetic ancestry group among unrelated HCHS/SOL participants

|  | Central American | | Cuban | | Dominican | | Mexican | | Puerto-Rican | | South American | |
| --- | --- | --- | --- | --- | --- | --- | --- | --- | --- | --- | --- | --- |
|  | < 60  N=819 | 60 +  N=112 | < 60  N=1297 | 60 +  N=382 | < 60  N=600 | 60 +  N=125 | < 60  N=2457 | 60 +  N=443 | < 60  N=1115 | 60 +  N=303 | < 60  N=500 | 60 +  N=112 |
| Genotype | | | | | | | | | | | | |
| apoE4- | 79.49 | 77.68 | 76.95 | 76.70 | 70.17 | 62.10 | 78.92 | 81.26 | 74.89 | 75.25 | 80.20 | 77.68 |
| apoE4+ | 20.51 | 22.33 | 23.05 | 23.30 | 29.83 | 37.90 | 21.08 | 18.74 | 25.11 | 24.75 | 19.80 | 22.32 |
| P-value (χ2) | 0.66 | | 0.92 | | 0.08 | | 0.26 | | 0.89 | | 0.55 | |
| Allele | | | | | | | | | | | | |
| E2 | 3.79 | 4.46 | 6.28 | 7.20 | 8.25 | 10.48 | 2.85 | 2.93 | 5.43 | 4.46 | 3.50 | 4.02 |
| E3 | 85.35 | 83.93 | 81.11 | 80.37 | 75.08 | 68.95 | 85.96 | 87.25 | 81.03 | 83.00 | 85.60 | 83.48 |
| E4 | 10.87 | 11.61 | 12.61 | 12.43 | 16.67 | 20.56 | 11.19 | 9.82 | 13.54 | 12.54 | 10.90 | 12.50 |
| P-value (χ2) | 0.82 | | 0.66 | | 0.13 | | 0.48 | | 0.48 | | 0.72 | |

**Supplemental Table 3**. ApoE allele frequency distribution (percent) by self-identified ancestry background

|  | Central American  N=886 | Cuban  N=1528 | Dominican  N=742 | Mexican  N=2897 | Puerto Rican  N=1385 | South American  N=562 | More than one  N=245 | NA  N=20 | P-value (χ^2^) |
| --- | --- | --- | --- | --- | --- | --- | --- | --- | --- |
| E2 | 4.01 | 6.28 | 8.29 | 2.87 | 5.05 | 4.09 | 6.32 | 7.50 |  |
| E3 | 84.99 | 81.68 | 74.39 | 86.08 | 81.23 | 84.79 | 81.22 | 80.00 |  |
| E4 | 11.00 | 12.04 | 17.32 | 11.05 | 13.72 | 11.12 | 12.45 | 12.50 | <0.0001 |
